# Heme (dys)homeostasis and liver disease

**DOI:** 10.3389/fphys.2024.1436897

**Published:** 2024-07-29

**Authors:** Tiago L. Duarte, Nicole Viveiros, Catarina Godinho, Delfim Duarte

**Affiliations:** ^1^ i3S–Instituto de Investigação e Inovação em Saúde, Universidade do Porto, Porto, Portugal; ^2^ IBMC–Instituto de Biologia Molecular e Celular, Universidade do Porto, Porto, Portugal; ^3^ Faculdade de Medicina da Universidade do Porto (FMUP), Porto, Portugal; ^4^ Serviço de Hematologia e Transplantação da Medula Óssea, Instituto Português de Oncologia do Porto Francisco Gentil, E.P.E. (IPO Porto), Porto, Portugal; ^5^ Departamento de Biomedicina, Faculdade de Medicina da Universidade do Porto (FMUP), Porto, Portugal

**Keywords:** heme, iron metabolism, liver disease, porphyria, ferroptosis, liver cancer, hemolysis, immune response

## Abstract

Heme is essential for a variety of proteins involved in vital physiological functions in the body, such as oxygen transport, drug metabolism, biosynthesis of steroids, signal transduction, antioxidant defense and mitochondrial respiration. However, free heme is potentially cytotoxic due to the capacity of heme iron to promote the oxidation of cellular molecules. The liver plays a central role in heme metabolism by significantly contributing to heme synthesis, heme detoxification, and recycling of heme iron. Conversely, enzymatic defects in the heme biosynthetic pathway originate multisystemic diseases (porphyrias) that are highly associated with liver damage. In addition, there is growing evidence that heme contributes to the outcomes of inflammatory, metabolic and malignant liver diseases. In this review, we summarize the contribution of the liver to heme metabolism and the association of heme dyshomeostasis with liver disease.

## 1 Introduction

Heme, a complex of iron with protoporphyrin IX, serves as a prosthetic group in several hemoproteins involved in oxygen transport and storage (hemoglobin and myoglobin), peroxide inactivation (catalase, peroxidases), electron transport, energy generation and chemical transformation (cytochromes), oxidation of tryptophan (tryptophan dioxygenases), among others (Ponka, 1999). In humans and other higher animals, both heme synthesis and degradation are highly regulated processes. Heme biosynthesis, mainly performed by developing erythroid cells and hepatocytes (which are responsible for 15% of the daily heme production) is a highly conserved process that involves eight enzymes, four of which are cytoplasmic, whereas the remaining four are mitochondrial (Ajioka et al., 2006). In both erythroid and nonerythroid tissues, heme biosynthesis is mainly regulated at the level of the first and rate-controlling enzyme, Aminolevulinic acid synthase (ALAS), albeit by different mechanisms. Tissue-specific regulation is ensured by the existence of 2 different genes for ALAS, one expressed ubiquitously (ALAS1) and the other expressed only in erythroid precursors (ALAS2), which are differentially regulated. ALAS2 is regulated by erythroid specific factors (Surinya et al., 1997) and by the interaction of IRE binding protein (IRP) with an iron regulatory element (IRE) in the 5′-untranslated region of ALAS2 mRNA. The IRE–IRP complex prevents translation of the ALAS2 mRNA, whereas addition of an iron–sulfur cluster (Fe/S) abrogates the ability of IRPs to bind to the IRE and allows translation to occur. This ensures that the rate-limiting step of erythroid heme production is controlled by iron availability (Wingert et al., 2005; Ajioka et al., 2006). Likewise, expression of ferroportin (FPN1), the unique cellular iron exporter, is mostly regulated by erythroid-specific factors, as erythroid precursors make use of an alternative upstream promoter to express FPN1 transcript that lacks the IRE and is thus not repressed in iron-deficient conditions (Zhang et al., 2009). As a result, in iron-depleted conditions, iron export from FPN1 increases, which further contributes to repress heme synthesis and erythropoiesis (Zhang et al., 2011). On the other hand, ALAS1 is regulated by the Peroxisome proliferator-activated receptor γ coactivator 1α (PGC-1α) (Handschin et al., 2005) and the hepatic heme synthesis is regulated by heme-mediated feedback inhibition, through inhibition of transcription of ALAS1 gene and translation of ALAS1 mRNA, destabilization of ALAS1 transcript, and inhibition of translocation of the ALAS1 protein precursor into the mitochondrial matrix (Ponka, 1999).

Heme breakdown is also a highly controlled process, regulated by Heme oxygenase (HMOX). Most of the iron-containing porphyrin that is degraded comes from the hemoglobin present in senescent erythrocytes that are phagocytosed by reticuloendothelial macrophages in the spleen and liver. During erythrophagocytosis, heme transport from the phagolysosome to the cytoplasm relies on SLC48A1 (also known as Heme-responsive gene-1, HRG1), a heme transporter that is highly expressed in reticuloendothelial macrophages (White et al., 2013). HRG1 deficiency in mice induces hemozoin formation due to heme accumulation into lysosomes, thus preventing heme recycling (Pek et al., 2019). The reaction catalyzed by HMOX leads to the release of iron, which is recycled, and the final products carbon monoxide (CO) and biliverdin, which is eventually reduced to the antioxidant bilirubin (Kikuchi et al., 2005). HMOX1, the inducible isoform of HMOX, is highly expressed in splenic macrophages and Kupffer cells (Bauer et al., 1998). Increased intracellular heme levels induce HMOX1 expression via transcription factor Nuclear factor erythroid 2-related factor 2 (NRF2), upon release of the heme-sensitive transcriptional repressor BTB domain and CNC homology 1 (BACH1) from stress response elements located in an enhancer region of the gene (Ogawa et al., 2001).

In addition, the liver plays an important role in recycling the heme iron of damaged erythrocytes. In mice, stress erythrophagocytosis is performed by a population of bone marrow-derived Ly-6C^+^ monocytes that home to the liver, where they differentiate into Ferroportin 1 (FPN1)-expressing macrophages, which deliver iron to hepatocytes (Theurl et al., 2016). The differentiation depends on growth factor Colony Stimulating Factor 1 (CSF1) and on NRF2 (Theurl et al., 2016), which is known to mediate the transcriptional activation of the FPN1 gene in macrophages (Marro et al., 2010).

In addition to regulating genes involved in its own biosynthesis or breakdown, heme regulates genes coding for globins, cytochromes, myeloperoxidase, and iron import/export proteins (Transferrin receptor, FPN1) (Chiabrando et al., 2014).

Whilst heme proteins are essential for a variety of vital physiological functions in the body, free heme is potentially cytotoxic due to the capacity of heme iron to promote the oxidation of cellular proteins, lipids and DNA (Chiabrando et al., 2014). The liver plays a major role in the regulation of circulating heme levels, by producing the two soluble scavengers of free hemoglobin and heme, haptoglobin (Schaer and Buehler, 2013; Schaer et al., 2013) and hemopexin, respectively. Haptoglobin binds to circulating hemoglobin, preventing its extravascular translocation, as well as its reaction with nitric oxide (NO) and peroxides, and the release of hemin (Schaer et al., 2013). Haptoglobin-hemoglobin complexes are endocytosed in liver macrophages through the hemoglobin scavenger receptor CD163 (Kristiansen et al., 2001). Hemopexin binds free heme forming hemopexin-heme complexes that are internalized by Low-density lipoprotein receptor-related protein (LRP)/CD91-expressing hepatocytes and the Kupffer cells (Hvidberg et al., 2005). These hepatic cell types catabolize heme, thus preventing heme-mediated oxidative stress and heme-bound iron loss, particularly in pathologic conditions associated with intravascular hemolysis (Tolosano et al., 2010). Besides hemopexin and haptoglobin, other circulating proteins produced by the liver are known to bind heme, thus preventing heme-mediated oxidative stress: albumin, high/low-density lipoprotein (LDL/HDL), and α1-microglobulin (Larsen et al., 2012).

**FIGURE 1:**
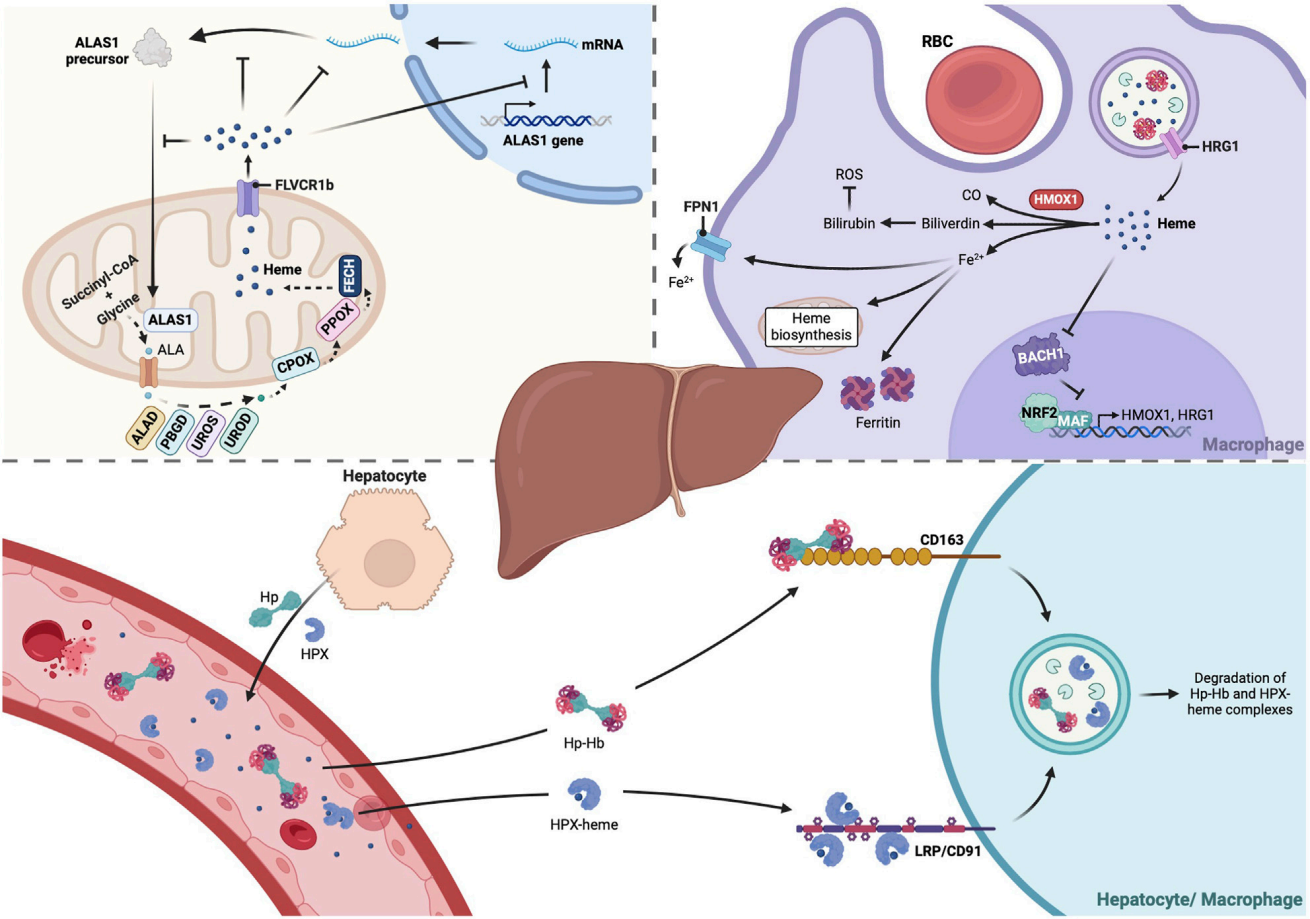
The liver plays a central role in heme metabolism. The liver is tightly involved in all stages of heme metabolism, from its biosynthesis to its breakdown, and recycling of heme iron. About 15% of heme daily production occurs in hepatocytes, where the heme biosynthetic pathway is mainly regulated by the isoform 1 of the rate-limiting enzyme ALAS (ALAS1), which is feedback regulated by heme. Moreover, reticuloendothelial macrophages of the liver are vital to clear senescent RBCs by phagocytosis. RBCs-derived heme is transported from the phagolysosome to the cytoplasm by HRG1, then catalyzed by HMOX and the resulting ferrous iron (Fe2+) is either retained in ferritin molecules or exported through FPN1 and recycled for production of new RBCs in the bone marrow. Hepatocytes are also responsible for production of Hp and HPX, which bind to Hb or free heme, respectively, targeting them to CD163^+^ liver macrophages or LRP/CD91^+^ hepatocytes and Kupffer cells. These molecular scavengers reduce heme oxidative reactivity and subsequent cytotoxicity, preventing organ damage. Created with BioRender.com. ALA, 5-aminolevulinic acid; ALAD, Aminolevulinic acid dehydratase; ALAS1, Aminolevulinic acid synthase-1; BACH1, BTB and CNC homology 1; CO, Carbon monoxide; CPOX, Coproporphyrinogen III oxidase; FECH, ferrochelatase; FLVCR1b, Feline leukemia virus subgroup C receptor-protein; FPN1, Ferroportin; Hb, Hemoglobin; HMOX1, heme oxygenase 1; Hp, Haptoglobin; HPX, Hemopexin; HRG1, Heme-responsive gene-1; NRF2, Nuclear factor erythroid 2-related factor 2; PBGD, Porphobilinogen deaminase; PPOX, Protoporphyrinogen oxidase; RBC, Red blood cell; ROS, Reactive Oxygen Species; UROD, Uroporphyrinogen decarboxylase; UROS, Uroporphyrinogen synthase.

In summary, the liver contributes significantly to heme synthesis and recycling, and the detoxification of circulating heme relies on proteins produced in the liver (Figure 1).

## 2 Heme biosynthesis gone wrong: hepatic and erythropoietic porphyrias

The porphyrias are a group of eight genetic diseases that result from defects in the different enzymatic steps of the heme biosynthetic pathway (Dickey et al., 2024): X-linked protoporphyria (XLP), d-aminolevulinic acid dehydratase (ALAD) deficiency porphyria (ADP), acute intermittent porphyria (AIP), congenital erythropoietic porphyria (CEP), porphyria cutanea tarda (PCT), hereditary coproporphyria (HCP), variegate porphyria (VP), and erythropoietic protoporphyria (EPP). The accumulation of specific enzyme substrates explains the clinical symptoms, which include either acute neurovisceral attacks or photosensitivity or both. Neurovisceral symptoms are due to neurotoxic effects of porphyrin precursors, whereas the photosensitivity is due to the fluorescent properties of porphyrins. Some authors have classified porphyrias according to their clinical symptoms as acute hepatic porphyrias (AHP) (AIP, VP, HCP, and ADP) and cutaneous, with the latter category comprising both blistering (PCT, CEP, VP, HCP) and nonblistering (EPP, XLP). Porphyrias are also traditionally divided into two categories, depending on the primary site of heme precursor overproduction: hepatic (ADP, AIP, PCT, HCP and VP) and erythropoietic porphyrias (XLP, CEP and EPP). For an up-to-date classification of the porphyrias and for insights on their clinical management, readers are advised to consult the excellent review of Dickey and colleagues (Dickey et al., 2024).

Regardless of whether they are classified as hepatic or erythropoietic, nearly all porphyrias relate in some way to the liver. Firstly, because some porphyrias are caused by liver damage. PCT, the most common type of porphyria, is in most cases (type I) an acquired deficiency of Uroporphyrinogen decarboxylase (UROD), the fifth enzyme in heme biosynthesis, caused by underlying liver diseases triggered by alcohol, iron overload, chlorinated hydrocarbons, oestrogens or viral hepatitis (Frank and Poblete-Gutierrez, 2010). Secondly, some porphyrias are associated with increased risk of primary liver cancer. This is the case of both PCT and AHP, as discussed below (Section 4). Thirdly, even erythropoietic porphyrias are known to damage the liver. EPP is caused by an inherited loss-of-function mutation in the gene for the final enzyme of heme biosynthesis, Ferrochelatase (FECH), whereas XLP patients carry gain-of-function pathogenic variants in erythroid-specific ALAS2, which encodes the first enzyme of heme synthesis. Erythropoietic protoporphyrias (EPP and XLP) are characterized by tissue accumulation of hydrophobic protoporphyrin, which can absorb energy from light and damage the endothelium and subcutaneous tissues through a process that is mediated by the production of reactive oxygen species (ROS). Patients are mostly affected by severe painful cutaneous phototoxicity after light exposure (Hussain et al., 2023). However, since protoporphyrin is excreted in the bile, some patients accumulate porphyrin-containing bile plugs, leading to severe cholestatic liver disease (Anstey and Hift, 2007). A recent study proposed the silencing of the hepatic bile acid-related nuclear farnesoid x receptor (FXR), which induces the expression of genes involved in heme biosynthesis, as a potential new therapeutic approach against EPP-associated cholestasis (Dean et al., 2023). Lastly, the treatment of some porphyrias may require liver transplantation (Lissing et al., 2024), either to treat the porphyria or its symptoms, as is the case of AIP (Lissing et al., 2021) or EPP (Anstey and Hift, 2007). Likewise, increasing the hepatic production of specific enzymes of the heme biosynthetic pathway via systemic messenger RNA therapy (e.g., hPBGD mRNA) is emerging as a potential treatment for porphyria patients (Jiang et al., 2018).

## 3 Heme, vascular occlusion, immune response and metabolic alterations in liver disease

**FIGURE 2:**
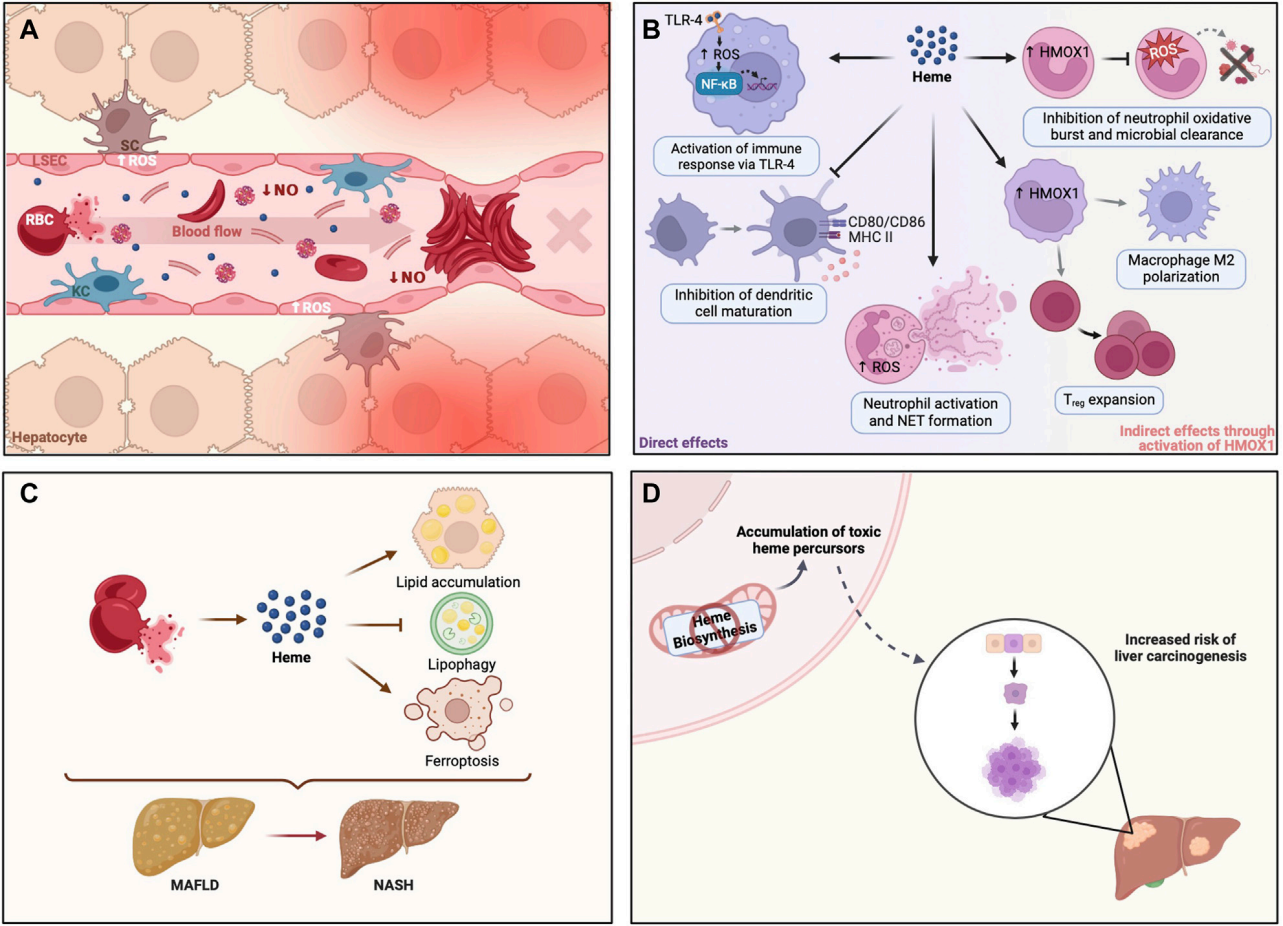
Heme dyshomeostasis is associated with liver disease. **(A)** In sickle cell disease, RBCs are more fragile and break apart during circulation, releasing significant amounts of heme and heme-enriched membrane microparticles. Such amounts of cell-free heme in circulation activate endothelial cells via ROS production and promote abnormal RBC adhesion, originating clumps that can block blood flow. This vascular occlusion may occur in liver sinusoids, preventing liver oxygenation and thus causing severe tissue damage. **(B)** Heme is a danger-associated molecular pattern (DAMP) that can modulate different cells in the immune compartment. Heme can activate innate immune receptors (e.g., TLR-4) and neutrophil NET formation, activating and amplifying inflammation. In turn, heme may inhibit dendritic cell maturation or modulate HMOX1 expression in monocytes, favoring T_reg_ expansion and macrophage M2 polarization. These anti-inflammatory responses are of the utmost importance in sterile liver inflammation or liver transplantation. Heme can also increase the expression of heme-inducible HMOX1 in immature neutrophils, inhibiting a proper oxidative burst in resulting mature neutrophils, thereby preventing host defense against pathogens targeting different organs, including the liver. **(C)** Cell-free heme, released during hemolysis, may promote metabolic liver disease by disruption of hepatic lipid metabolism. Hemolysis has been shown to promote lipid accumulation and block intracellular breakdown of lipid droplets by lipophagy, resulting in liver steatosis, a well-known trait of MAFLD. Heme-iron may also initiate lipid peroxidation, mediating the death of hepatocytes by ferroptosis, which potentially plays a role in the progression of MAFLD to NASH. **(D)** Enzymatic defects in the heme biosynthetic pathway result in metabolic disorders known as porphyrias, due to accumulation of different heme precursors (i.e., porphyrins), some of which are genotoxic. Their accumulation is associated with an increased risk of carcinogenesis. Created with BioRender.com. HMOX1, heme oxygenase 1; KC, Kupffer cell; LSEC, Liver sinusoidal endothelial cell; MAFLD, Metabolic dysfunction-associated fatty liver disease; NASH, Non-alcoholic steatohepatitis; NET, Neutrophil extracellular trap; NF-kB, Nuclear factor kappa B; NO, nitric oxide; RBC, Red blood cell; ROS, Reactive Oxygen Species; SC, Stellate cell; TLR-4, Toll-like receptor 4; T_reg_, Regulatory T cell.

Liver damage is a common finding in patients with sickle cell disease (SCD), mostly due to vascular occlusion from intrahepatic sickling of erythrocytes with concomitant acute ischemia (Theocharidou and Suddle, 2019). In SCD, hemolysis results in the release of large quantities of free heme and heme-laden erythrocyte membrane microparticles into the circulation (Figure 2A). This triggers endothelial cell activation, via production of ROS, and abnormal red blood cell adhesion (Camus et al., 2015; An et al., 2023). In addition, free plasma hemoglobin depletes circulating levels of NO, which causes smooth muscle contractions and vasoconstriction (Piccin et al., 2019). Another consequence of chronic hemolysis in SCD is the development of pigment gallstones, whereas viral hepatitis and iron overload may develop secondary to multiple blood transfusions (Theocharidou and Suddle, 2019). Additionally, some SCD patients present autoimmune hepatitis (Lynch et al., 2023).

Free heme is a well-established danger-associated molecular pattern (DAMP), which can initiate immune responses upon binding to Toll-like receptor 4 (TLR4) (Canesin et al., 2020). However, there is accumulating evidence that hemolysis can modulate immune cell differentiation and function in different ways (reviewed by Zhong and Yazdanbakhsh, 2018). Via the production of ROS, free heme can induce the formation of neutrophil extracellular traps (NETs), which contributes to the vaso-occlusive crises in SCD patients. Heme can also induce HMOX1 in immature neutrophils, which inhibits the oxidative burst and impairs the capacity of leukocytes to destroy pathogenic agents (Evans et al., 2015). Hemolysis can also favor anti-inflammatory immune cell polarization by inhibiting dendritic cell maturation required for effector T-cell responses (Zhong and Yazdanbakhsh, 2018), and induce the differentiation of liver macrophages into anti-inflammatory erythrophagocytes (Pfefferlé et al., 2020), which was shown to provide protection against sterile liver inflammation (Pfefferlé et al., 2021). Likewise, hemolysis may promote regulatory T-cell (T_reg_) expansion through modulation of HMOX1 expression in nonclassical monocytes (Zhong and Yazdanbakhsh, 2018; Figure 2B).

The heme-inducible enzyme HMOX1 was reported to play a protective role in hepatic ischemia–reperfusion injury (IRI) following orthotopic liver transplantation. Low HMOX1 expression by liver macrophages correlates with hepatocellular death and worse patient survival (Nakamura et al., 2017). Mechanistically, HMOX1 prevents macrophage M1 polarization (Zhang et al., 2018) and TLR4-driven inflammatory responses (Rao et al., 2015). Another study reported the ability of HMOX1 to modulate T_reg_ expansion and to inhibit infiltration of CD4^+^ and CD8^+^ cells in transplanted livers (Sun et al., 2011).

Hemolysis may enhance liver damage by promoting metabolic dysfunction-associated fatty liver disease (MAFLD) (Figure 2C). Using a mouse model of acute intravascular hemolysis, Rayego-Mateos et al. (2023), demonstrated that heme disturbs lipid metabolism and promotes liver steatosis. Specifically, hemolysis exacerbates lipid accumulation and blocks the lipophagy pathway. On the other hand, heme-inducible enzymes and products of heme breakdown with antioxidant properties seem to be protective. The catabolism of heme mediated by HMOX produces biliverdin, which is reduced to bilirubin by Biliverdin reductase (BVR) (O’Brien et al., 2015). HMOX1 expression is increased in non-alcoholic steatohepatitis (NASH) patients (Malaguarnera et al., 2005) and an *in vitro* study suggests that HMOX1 may have a protective role by suppressing endoplasmic reticulum stress in hepatocytes (Li X. et al., 2020). Several studies have shown that serum bilirubin levels are inversely correlated with the prevalence of MAFLD in the general population (Kwak et al., 2012; Han et al., 2024), and with less severe liver disease among MAFLD patients (Kumar et al., 2012; Puri et al., 2013; Salomone et al., 2013; Han et al., 2024), which was repeatedly hypothesized to be due to the antioxidant effect of bilirubin. However, this was never demonstrated experimentally and remains a speculation. In fact, a study by Stec et al. (2016) demonstrated that bilirubin inhibits lipid accumulation in mice through direct binding to Peroxisome proliferator-activated receptor α (PPARα). Moreover, Biliverdin reductase A (BVRA), the enzyme that reduces biliverdin IXα to bilirubin IXα, was also shown to prevent hepatic lipid accumulation in a study using mice with liver-specific BVRA KO fed with high-fat diet, through inhibition of Glycogen synthase kinase (GSK) 3β and activation of PPARα (Hinds et al., 2016).

In addition to the effects on hepatic steatosis, hepatocellular free heme may also promote the progression of MAFLD to NASH by catalyzing Fenton-like reactions, lipid peroxidation and ultimately hepatocellular death by ferroptosis (Zhao et al., 2023). Ferroptosis is an iron-dependent and lipid peroxidation-mediated nonapoptotic cell death that was shown to be the initiator of inflammation in the methionine-choline deficient diet mouse model of NASH (Tsurusaki et al., 2019; Li D. et al., 2020; Qi et al., 2020). Taking all the evidence above into consideration, reducing free heme may constitute a therapeutic approach in the treatment of metabolic liver disease (Zhao et al., 2023).

## 4 Heme and liver cancer

The AHP, especially AIP, are associated with a marked increased risk of primary liver cancer, mainly hepatocellular carcinoma (HCC) (Baravelli et al., 2017; Saberi et al., 2021; Lissing et al., 2022; Figure 2D). Notably, HCC in AHP occurs in the absence of cirrhosis, unlike other chronic liver diseases (Saberi et al., 2021). The pathogenesis of hepatocarcinogenesis in AHP remains unknown but it may be related to the intrahepatic accumulation of 5-Aminolevulinic acid, which is pro-oxidant and genotoxic (Onuki et al., 2002). Alternative hypotheses such as a loss of antioxidant effects due to heme deficiency, or direct or indirect effects of mutations in heme metabolism genes have also been proposed (Peoc’h et al., 2019). This is in contrast with PCT patients, who also show increased risk of primary liver cancer (Baravelli et al., 2019), but whose porphyria is concomitant to an underlying liver disease that increases the risk of cirrhosis and primary liver cancer. Likewise, chronic exposure of laboratory rodents to some chemicals, drugs and pesticides that cause hepatic porphyria is associated with liver carcinogenesis (reviewed by Smith and Foster, 2018).

Heme per se is also believed to be carcinogenic, at least in colorectal cancer. There is wide evidence that heme iron is the critical component of red meat that promotes colorectal carcinogenesis (Seiwert et al., 2020). Excess heme iron may promote carcinogenesis by: favoring ROS production and the oxidation of DNA, lipids and proteins; suppressing TP53 activity; modulating immune cell function, inflammation, and gut dysbiosis (Gamage et al., 2021).

Cancer cells are reported to have greater activity of heme-containing proteins and increased heme content, which may be partly explained by the upregulation of the main enzymes in heme synthesis (e.g., ALAS) (Fiorito et al., 2020; Gamage et al., 2021). In other cases, such as in acute myeloid leukemia, heme biosynthesis is downregulated (Lin et al., 2019). The heme-iron exporter Feline leukemia virus subgroup C receptor-related protein 1 (FLVCR1) is also over-expressed in several tumors (Peng et al., 2018; Russo et al., 2019), possibly to establish a balance in intracellular heme homeostasis. In fact, both increased heme synthesis and increased heme export were shown to control the energetic metabolism of cells with high-energy demand, such as tumor cells (Fiorito et al., 2021). Notably, in the particular case of HCC, analyses of publicly available RNAseq data at the “The Cancer Genome Atlas” (TCGA) showed that: a) FLVCR1 mRNA expression is significantly increased in HCC when compared with normal liver tissue; b) FLVCR1 amplification or mRNA upregulation are observed in 21% of HCC cases; c) FLVCR1 mRNA expression is significantly associated with HCC disease status, histological grade, and vascular invasion; and d) higher expression of FLVCR1 is associated with poor overall survival in HCC (Shen et al., 2018; Tang et al., 2020; Wei et al., 2020). Likewise, analyses of tissue samples obtained from the Human Protein Atlas showed that FLVCR1 protein is strongly detected in HCC tissue, but not in normal liver tissue (Shen et al., 2018; Wei et al., 2020). While FLVCR1 is emerging as a new significant predictor of prognosis and a useful diagnosis marker in HCC, its role in the pathophysiology of HCC remains unknown.

Increased expression of proteins involved in heme uptake has also been implicated in cancer (Hooda et al., 2013). HRG1 may contribute to cancer cell invasiveness (Fogarty et al., 2014) by regulating the activity of vacuolar-(H(+)) ATPase (V-ATPase), which is essential for endosomal acidification and receptor trafficking in mammalian cells (O’Callaghan et al., 2010). Although this has not been addressed specifically in the context of liver cancer, HRG-1 may also represent a target for disrupting V-ATPase activity and decrease the metastatic potential of cancer cells (Fogarty et al., 2014).

Finally, excess iron may promote cell death by ferroptosis, which is a mechanism of tumor suppression that has been implicated in the action of clinical agents used to treat HCC (e.g., sorafenib). There is increasing evidence that activating ferroptosis may potently inhibit the growth of HCC cells (Chen et al., 2022; Jiang et al., 2024). The fact that cancer cells exhibit higher levels of heme could potentially be explored in the search for a novel therapeutic strategy for HCC, which is among the leading causes of cancer-related mortality worldwide. However, the contribution of heme-bound iron to ferroptosis remains unclear.

## 5 Conclusion

Heme is a key component of cellular respiration and function. Heme biosynthesis is therefore tightly regulated and changes in enzymatic activity of the pathway are associated with disease. Heme also plays an essential role in oxygen transport and its uncontrolled release (e.g., hemolysis) is associated with vascular damage and malfunction and tissue oxidation. An emerging topic of research is how cellular heme impacts on cancer cell proliferation and death. This is particularly relevant in tissues enriched for heme proteins, such as the liver. Its study may reveal cancer-specific cell dependencies related with heme production and heme handling that are still unknown.
